# Biocontrol activity of *Bacillus halotolerans* strain Pl7 against *Botryosphaeria dothidea* causing apple postharvest decay and potential mechanisms

**DOI:** 10.3389/fmicb.2022.1058167

**Published:** 2023-01-04

**Authors:** Hongbo Yuan, Mengjia Yuan, Bingke Shi, Zhuoni Wang, Tianxiang Huang, Jiahong Zhu, Hui Hou, Li Wang, Hongtao Tu

**Affiliations:** Zhengzhou Fruit Research Institute, Chinese Academy of Agricultural Sciences, Zhengzhou, China

**Keywords:** *Botryosphaeria dothidea*, biocontrol activity, *Bacillus halotolerans*, transcriptomics, apple ring rot

## Abstract

Apple ring rot, one of the most common apple postharvest diseases during storage, is caused by *Botryosphaeria dothidea*. Fungicide application is the most widely used method to control this disease, but the increasing environmental and food safety concerns greatly limit their use. The present study aimed to examine the biocontrol activity and underlying action mechanism of *Bacillus halotolerans* strain Pl7 against *B. dothidea*. The results revealed that *B. halotolerans* strain Pl7 exhibited strong inhibitory activity against *B. dothidea* by 69% *in vitro*. The culture filtrate of strain Pl7 possessed cellulase, β-1, 3-glucanase, protease activity and mediated the antifungal activity against *B. dothidea*. Further analysis demonstrated that culture filtrate of strain Pl7 could cause cell membrane permeabilization of *B. dothidea*. Apple fruit suffering from ring rot induced by a carbendazim (CBZ)-sensitive or -resistant *B. dothidea* isolate was much suppressed after being treated with strain Pl7, maintaining postharvest quality. The ability of strain Pl7 to swiftly colonize and thrive in apple fruit wounds was demonstrated by a re-isolation assay. Additional transcriptome studies of untreated and treated apple fruit with strain Pl7 revealed that strain Pl7 mostly changed the expression of genes functioning in plant secondary metabolite biosynthesis and plant-pathogen interaction. In light of these outcomes, the underlying antagonistic mechanism was investigated, and *B. halotolerans* strain Pl7 was identified as a promsing microbial biocontrol agent against apple postharvest decay.

## Introduction

Apple ring rot, one of the postharvest infections that leads to great quantity and quality losses during apple storing, is caused by *Botryosphaeria dothidea* (Huang et al., 2021; Sun et al., 2022). In addition to infecting apple, *B. dothidea* infects other fruit tree species, such as pear, sweet cherry, and kiwifruit (Zhai et al., 2014; Zhang et al., 2019b; Wang et al., 2021). Besides, the widely planted cultivar in China, ‘Fuji’ cultivar, is very susceptible to apple ring rot (Li et al., 2013). Control is difficult due to the disease’s latent infection feature (Liu et al., 2011). The most popular strategy for controlling apple ring rot is pre-harvest fungicide application, such as carbendazim (CBZ) (Wang et al., 2022). However, environmental and food safety concerns, and the emergence of fungicide-resistant isolates, such as CBZ-resistant *B. dothidea* strains, severely limit the application of fungicides (Chen et al., 2021; Wang et al., 2022). As a result, there is a greater demand for alternative or supplementary disease control approaches.

A particularly appealing and widely used disease management strategy is endophyte-based biocontrol (Dubey et al., 2020). Previous research showed that a variety of microorganisms such as *Streptomyces rochei* (strain A-1), *Streptomyces lavendulae* (strain Xjy), *Meyerozyma guilliermondii* (strain Y-1), *B. amyloliquefaciens* (strain PG12), *Paenibacillus polymyxa* (strain APEC136), *B. subtilis* (strain 9,407), *B. atrophaeus* (strain J-1), and *B. velezensis* (strain P2-1), have been as biocontrol agents in controlling apple postharvest rot (Chen et al., 2016; Gao et al., 2016; Kim et al., 2016; Zhang et al., 2016; Fan et al., 2017; Mu et al., 2020; Huang et al., 2021; Yuan et al., 2022a), in part by preventing *B. dothidea* mycelial growth, secreting antifungal compounds, or triggering host defensive reactions.

*Bacillus halotolerans* is a possible biocontrol agent for plant pathogens. For example, *B. halotolerans* strain proved successful in suppressing strawberry grey mold caused by *Botrytis cinerea* (Wang et al., 2021). *B. halotolerans* was also found to greatly inhibit *Fusarium oxysporum* f. sp. *radicis*-*lycopersici* mycelical growth (Slama et al., 2019). However, no earlier research has described the utilization of *B. halotolerans* strains for the biocontrol of apple postharvest decay. In addition, the underlying mechanisms of *B. halotolerans* against pathogens remain largely unknown.

This study attempted to assess the biocontrol effectiveness of *B. halotolerans* strain Pl7, collected from the bark of an apple branch, against *B. dothidea*. Additionally, a potential antagonistic mechanism of *B. halotolerans* strain Pl7 against *B. dothidea*-caused apple ring rot was investigated.

## Materials and methods

### Endophytic bacterium and fungal pathogen

Endophytic bacterium strain Pl7 was previously isolated from the bark of apple (variety named “Kitanosach”) branch at Zhengzhou, Henan province, China in July 2021 by Yuan et al. (2022a) and cultured on LB solid medium (Peptone 10 gL^−1^, Yeast extract 5 gL^−1^, Sodium chloride 10 gL^−1^, Agar 15 gL^−1^) at 28°C.

CBZ-sensitive *B. dothidea* strain Bd220 (β-tubulin Genbank accession number: MZ197998) and CBZ-resistant *B. dothidea* strain Bd7 (β-tubulin Genbank accession number: MZ197999) were used in this study and cultured on potato dextrose agar (PDA) (Potato extracts 200 gL^−1^, Glucose 20 gL^−1^, Agar 15 gL^−1^) in Petri plates at 25°C (Wang et al., 2022).

### Dual culture assay

The dual culture assay based on a previous study (Yuan et al., 2022a) was used to test the antagonistic activity of strain Pl7 against pathogenic pathogens *in vitro*. A mycelial plug of the pathogen (*B. dothidea*) with 0.5 cm in diameter was inserted in the center of the Petri dish after 3 μl of overnight cultured-strain Pl7 [1 × 10^8^ colony forming units (CFU) mL^−1^] were seeded on PDA medium on either side (2 cm from the center). Mycelial colony diameter was recorded at 25°C, 6 days post inoculation (dpi). The PDA medium only inoculated with mycelial plug was used as control (CK). The experiment was performed three times, and each time had three replicates. Inhibition = (colony diameter of CK − colony diameter of treated)/(colony diameter of CK) × 100%. Hyphal morphological characteristics were observed under an ultra-depth three-dimensional microscope (KEYENCE, Japan) at 2 days after dual culture. The experiment was performed three times, and each time examined at least 10 hyphae.

### Identification of strain Pl7

A prior study was used to assess the physiological and biochemical characteristics of strain Pl7 (Holt et al., 1994). This strain was molecularly identified based on 16S rDNA, *gyrA*, and *rpoB* sequencing with primers (Supplementary Table S1; Yuan et al., 2022a). The PCR amplification procedure was performed as described previously (Yuan et al., 2022a). The PCR results were sequenced by Sangon Biotech Co., Ltd., Shanghai, China, and the obtained sequences were compared to the NCBI nucleotide database to get the homologous sequences. The MEGA 7.0 software was used to generate phylogenetic trees using the maxium likelihood method with 1,000 bootstrap iterations.

### Antifungal activity of strain Pl7 culture filtrate against *Botryosphaeria dothidea* mycelial development

Following a 3 days culture in LB broth medium (Peptone 10 gL^−1^, Yeast extract 5 gL^−1^, Sodium chloride 10 gL^−1^) at 28°C, 200 rpm/min, the samples were run through a 0.22 μm pore size filter to remove bacteria for getting the culture filtrate. The resulting culture filtrate was mixed with PDA medium at the highest culture filtrate concentrations of 5% or 10% (v/v). A *B. dothidea* plug with 0.5 cm in diameter was inoculated on each plate. The diameter of the mycelial colony was measured at 25°C, 6 dpi. The experiment was performed three times, and each time had three replicates.

### Secreted enzyme activity analyses

Hydrolytic enzyme activity of *B. halotolerans* strain Pl7, including β-1, 3-glucanase, cellulase, and protease activity, was assessed by incubating strain Pl7 on aniline blue medium, carboxymethylcellulose (CMC) medium, and skim milk medium on Petri dishes (Yuan et al., 2022a). After 3 dpi at 28°C, we observed whether there was a transparent circle around the bacterial colony.

### PCR amplification of antibiotic biosynthesis genes from strain Pl7

Total DNA was isolated from overnight cultured-strain Pl7 with DNA extraction kit (Solarbio, Beijing, China) according to the manufacturer’ procedure. Target antibiotic genes and primers were listed in Supplementary Table S1 (Lee et al., 2016; Kim et al., 2017). The PCR amplification procedure was performed as described previously (Lee et al., 2016; Kim et al., 2017). The PCR products were sequenced by Sangon Biotech Co., Ltd., Shanghai, China.

### Fluorescence observation

*B. dothidea* was inoculated into a potato dextrose broth (PDB) (Potato extracts 200 gL^−1^, Glucose 20 gL^−1^) medium for 2 days at 180 rpm/min, 25°C. Afterwards, *B. dothidea* hyphae were collected and treated with culture filtrate of *B. halotolerans* strain Pl7 for 24 h. LB broth medium treatment was used as CK. Hyphae were mixed with SYTOX green (1 μM; Biomart, Beijing, China) for 5 min in the dark. After staining, hyphae were rinsed five times with sterile water, and examined by using a Leica Laser Confocal Microscopy (TCS SP5, Germany) under 488 nm excitation and 538 nm emission.

### Antifungal activity of *Bacillus halotolerans* strain Pl7 against *Botryosphaeria dothidea* in apple fruit

The antifungal activity of *B. halotolerans* strain Pl7 to control *B. dothidea* growth in apple (Fuji) fruit was evaluated based on a method reported previously (Yuan et al., 2022a). Each apple (Fuji) was wounded twice with a sterile borer to produce the hole with 0.3 cm deep and 0.5 cm wide. Each hole was inoculated with 40 μl of strain Pl7 cell suspension (1 × 10^8^ CFU mL^−1^) with pipette. The negative control was sterile water treatment, while the positive control was the fungicide CBZ (0.8 gL^−1^; Jiangsu Longdeng Chemical Co., Ltd. Jiangsu, China) treatment with the same procedure as strain Pl7 treatment. After dry, fruit wounds were then inoculated with *B. dothidea* mycelial plugs (diameter: 0.5 cm) from the edge of 5-day-old colony. After that, the fruit were kept at 25°C with an 85% relative humidity in a climate box. At 3 and 5 dpi, a Vernier caliper was used to measure the length of the disease lesion. Disease incidence = the number of diseased sites/the total inoculation sites × 100%. This experiment was repeated three independent times and each time contained five fruits with 10 inoculation sites.

### Effect of *Bacillus halotolerans* strain Pl7 on apple fruit natural decay

Healthy and equal size apple (Fuji) fruits, collected from an orchard with severe ring rot disease, were used for assay. The fruits were dipped in 1 × 10^8^ CFU mL^−1^ cell suspension for 2 min and then naturally air dried. LB broth medium treatment was used as control. After treatment, the fruits were put in sterilized plastic boxes and kept at 20°C with an 80% relative humidity in an incubator. At the storage time points (21, 28, and 35 days), the number of rotted fruits was recorded. This assay was carried out twice, with three duplicates in each time. Each replicate consisted of 10 fruits.

### *Bacillus halotolerans* strain Pl7 colonization in apple fruit wounds

*B. halotolerans* strain Pl7 to colonize apple fruit was assessed based on the method reported previously (Yuan et al., 2022a). As described above, apple fruits were punctured by using a 0.5-cm sterile borer, and each hole (0.3 cm deep and 0.5 cm wide) was inoculated with strain Pl7 cell suspension (40 μl, 1 × 10^8^ CFU mL^−1^). Fruit tissues (around 0.6 g) were collected with a 0.7 cm core border at 0 (3 h after inoculation) to 9 dpi. After that, the tissue was pulverized with a grinder. 0.1 ml of each dilution was placed onto LB medium on Petri dishes after serial 10-fold dilution. Bacterial colonies in each Petri dishes were enumerated at 28°C, 2 dpi and converted to how many of colonies per wound. This test was carried out twice, with three duplicates in each time. For analysis, colony densities were converted as log_10_ CFU/wound.

### Transcriptome analysis

Healthy apples (Fuji) were dipped in cell culture of *B. halotolerans* strain Pl7 (1 × 10^8^ CFU mL^−1^) for 2 min, and about 1 *g* of fruit epidermal tissue from three apples was taken at 2 dpi to determine changes in overall gene expression pattern in apple fruit after strain Pl7 treatment. The assay was repeated three times. As a control, the LB broth treatment was used. Total RNA was extracted by using Trizol kit based on the instructions provided (TransGen Biotech, Beijing, China). Shanghai Personalbia Gene Technology Co., Ltd. performed the transcriptomic sequencing and analysis. Genes with a fold change [Log_2_ (fold change)] less than 1 and an adjusted *p*-value less than 0.05 were considered as differentially expressed genes (DEGs). The topGO software and the cluster profile package were used to perform gene ontology (GO) annotations and KEGG pathway enrichment analyzes, respectively (Alexa and Rahnenfuhrer, 2010; Yu et al., 2012).

### qRT-PCR assay

The qRT-PCR experiment was used to verify the accuracy of the RNA-seq data. Apple fruit samples were prepared and collected in the same manner as previously indicated for total RNA extraction. First-stand cDNA was created based on the instructions provided (Monad, Suzhou, China). The primers mentioned in Supplementary Table S1 were used in a qRT-PCR experiment conducted on a Roche PCR apparatus with a kit (Monad, Suzhou, China). The reference gene was elongation factor 1-a (*EF1a*). Six randomly selected DEGs were analyzed based on the 2^−ΔΔCt^ technique (Livak and Schmittgen, 2001). Three replicates were used in each of the two assay sets.

### Apple postharvest quality parameters assay

Freshly picked apples (Fuji) were dipped in strain Pl7 cell suspension (1 × 10^8^ CFU mL^−1^) for 2 min and then stored at 20°C in a transparent plastic in a incubator to evaluate the impact of strain Pl7 on the postharvest quality of apples. LB-treated apples were used as the CK. According to the previous study (Yuan et al., 2022a), four characteristics were assessed: soluble sugar content, titratable acidity, ascorbic acid content, and fruit hardness. The experiment was repeated twice and each test consisted of three replicates, with three fruit samples in each replicate.

### Statistical analysis

All data were analyzed by using DPS package. Following a Shapiro–Wilk test for normality, one-way ANOVA with Duncan’s multiple range test or Student’s *t*-test (*p* < 0.05) was used to determine significant differences between CK and treatments. All data are expressed as mean ± standard deviation (SD) of at least three biological replicates.

## Results

### Antagonistic activity of strain Pl7 against pathogen growth

**Figure 1:**
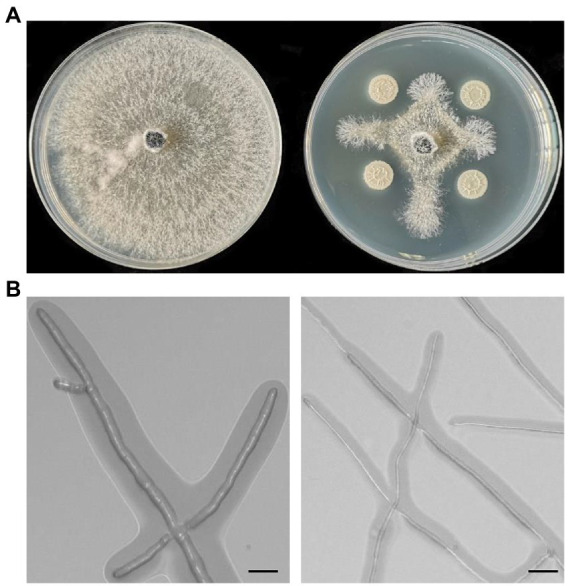
Strain Pl7 inhibition of *B. dothidea* mycelial development. **(A)** *B. dothidea* colony morphology at 6 dpi on PDA medium. **(B)** *B. dothidea* hyphal morphology 2 days after conflict with strain Pl7. *B. dothidea* CK is on the left. Scale Bar = 10 μm.

The dual culture assay showed that strain Pl7 had substantial antagonistic action against *B. dothidea*, inhibiting about 69% of mycelial development, with a 0.57 cm inhibitory zone (Figure 1A). Additional findings showed that strain Pl7-treated hyphae were unusually stretched and distorted with tenuous and ruptured compared to control hyphae (Figure 1B). These findings demonstrated that, collectively, strain Pl7 had strong antifungal activity against *B. dothidea in vitro*.

### Identification of strain Pl7

**Figure 2:**
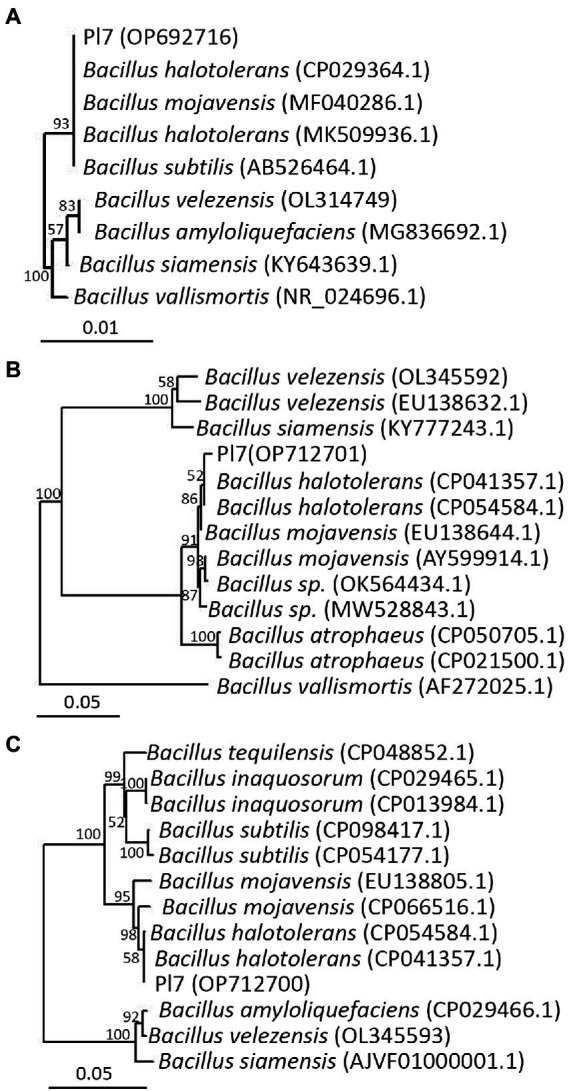
Based on 16S rDNA **(A)**, *gyrA* **(B)**, and *rpoB* **(C)** sequences, phylogenetic trees of strain Pl7 and its relatives were performed.

Supplementary Table S2 highlighted the biochemical and physiological characteristics of strain Pl7, revealing that it belonged to the genus *Bacillus*. When combined with closely comparable sequences, phylogenetic trees based on the 16S rDNA (accession number: OP692716), *gyrA* (accession number: OP712701), or *rpoB* (accession number: OP712700) sequences of strain Pl7 demonstrated that the strain Pl7 was more closely related to *B. halotolerans* (16s rDNA accession number: CP029364.1, MK509936.1; *gyrA* accession number: CP041357.1, CP054584.1; *rpoB* accession number: CP041357.1, CP054584.1; Figures 2A–C). As a result, strain Pl7 was identified as *B. halotolerans*.

### *Bacillus halotolerans* strain Pl7 culture filtrate inhibited *Botryosphaeria dothidea* mycelial growth

**Figure 3:**
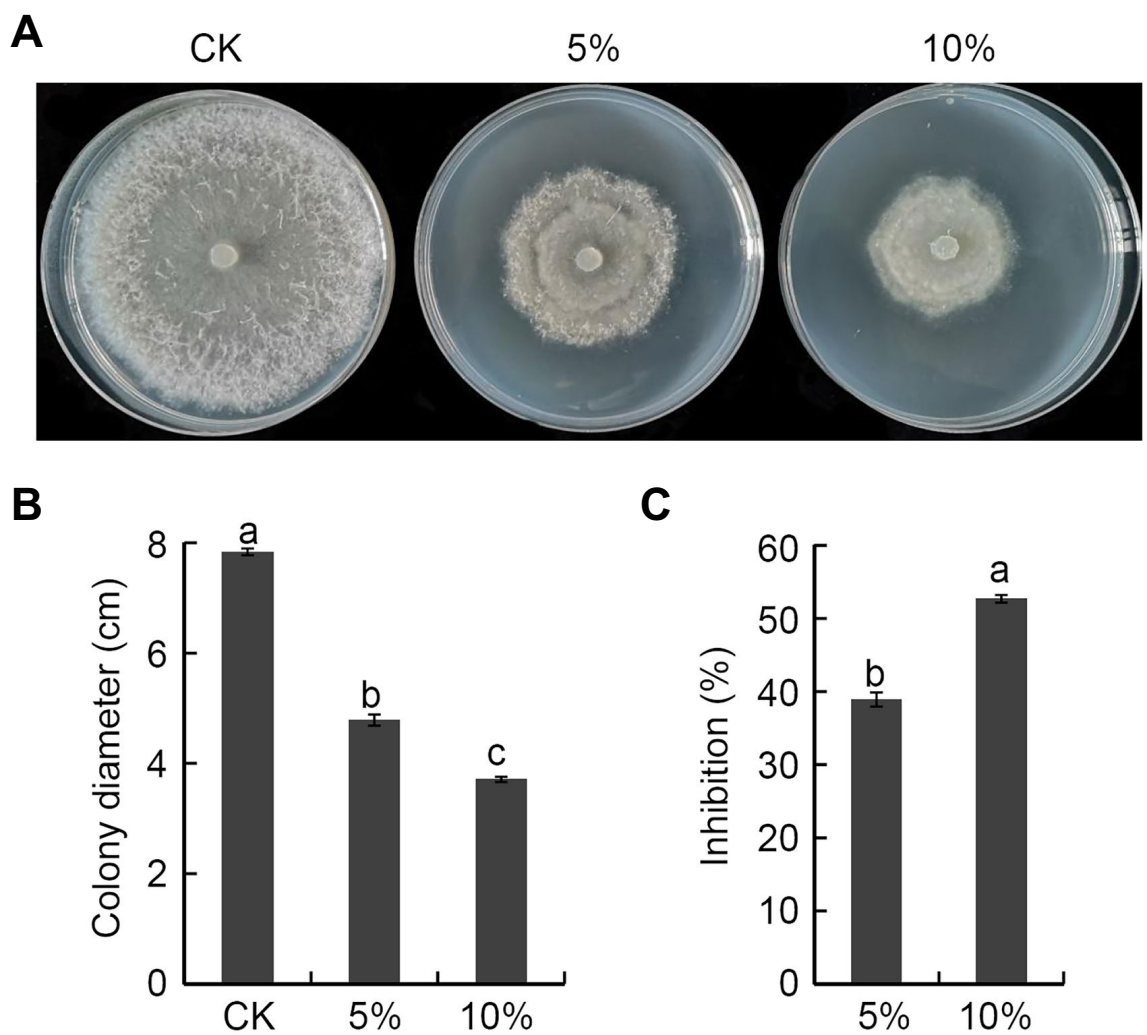
The impact of *B. halotolerans* strain Pl7 culture filtrate on *B. dothidea* mycelial growth. **(A)** *B. dothidea* colony morphology at 6 dpi on PDA medium with 5% or 10% culture filtrate of strain Pl7. **(B)** Measurements on the colony diameter of *B. dothidea*. Each dataset is expressed as the mean ± SD of three biological replicates. Different lowercase letters denote significant differences (*p* < 0.05; Duncan’s multiple test) between treatments and CK. **(C)** Rate of inhibition. Each dataset is expressed as the mean ± SD of three biological replicates. Different lowercase letters denote significance (*p* < 0.05; Student’s *t*-test) between treatments. The experiment was performed independently three times with similar results.

*B. dothidea* mycelial plugs were inoculated on a PDA medium containing 5 or 10% of the culture filtrate of *B. halotolerans* strain Pl7 to examine if it would exhibit antagonistic activity. The outcome showed that strain Pl7 culture filtrate greatly reduced the mycelial growth of *B. dothidea* (Figures 3A,B). The inhibition by 5 and 10% strain Pl7 culture filtrate, respectively, was 39 and 53% (Figure 3C). This result suggested that strain Pl7 culture filtrate had inhibition activity toward *B. dothidea*.

### Hydrolytic enzyme activity of *Bacillus halotolerans* strain Pl7

**Figure 4:**
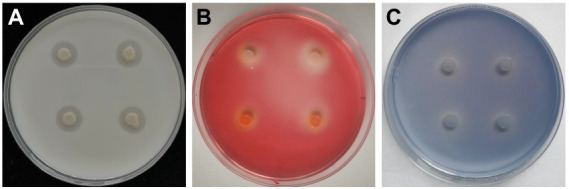
Hydrolytic enzyme activity of *B. halotolerans* strain Pl7. Protease **(A)**, cellulase **(B)**, and β-1, 3-glucanase **(C)** activity of *B. halotolerans* strain Pl7 was based on whether there was a transparent circle around the bacterial colony grown on skim milk medium, CMC medium and aniline blue medium, respectively. The pictures were taken at 3 dpi at 28°C.

The results revealed that strain Pl7 formed distinct translucent circles on different medium (Figures 4A–C), indicating protease, cellulase, and β-1, 3-glucanase activity in strain Pl7 produced enzymes or metabolites.

### PCR amplification of antibiotic biosynthesis genes from *Bacillus halotolerans* strain Pl7

**Figure 5:**
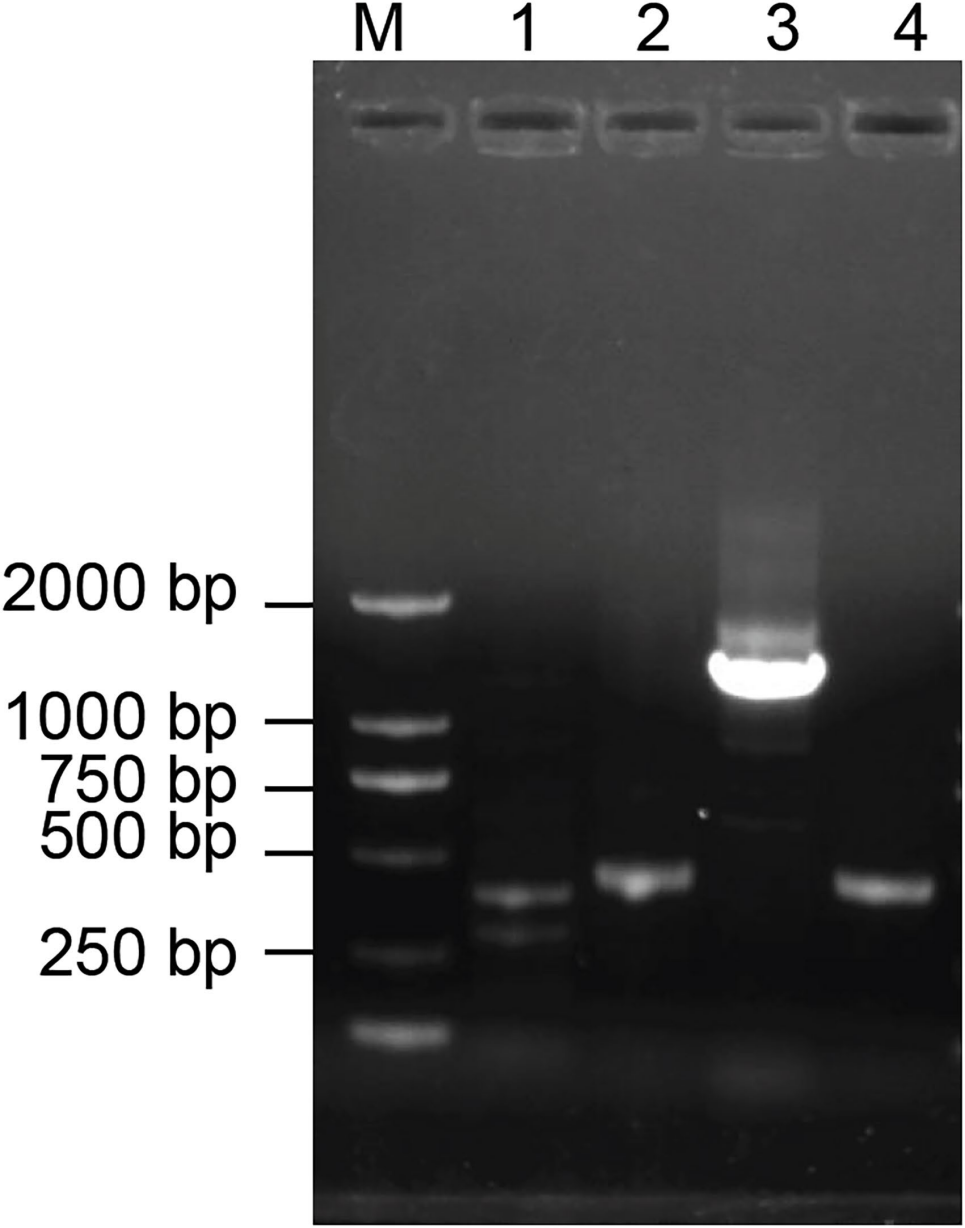
PCR analysis of antibiotic biosynthesis genes in strain Pl7. M: Trans 2 K DNA ladder, 1: *ituD*, 2: *srf*,3: *fen*, 4: *ituC*.

Lipopeptide is one of the major antimicrobial compounds secreted by *Bacillus* species. The result of PCR amplification showed that expected size of PCR products associated with *ituD*, *srf*, *fen*, and *ituC* were amplified from strain Pl7 (Figure 5). Further sequencing analysis confirmed the result that strain Pl7 harbored gene clusters involved in the synthesis of iturin, surfactin, and fengycin.

### Effect of *Bacillus halotolerans* strain Pl7 culture filtrate on *Botryosphaeria dothidea* hyphae membrane permeability

**Figure 6:**
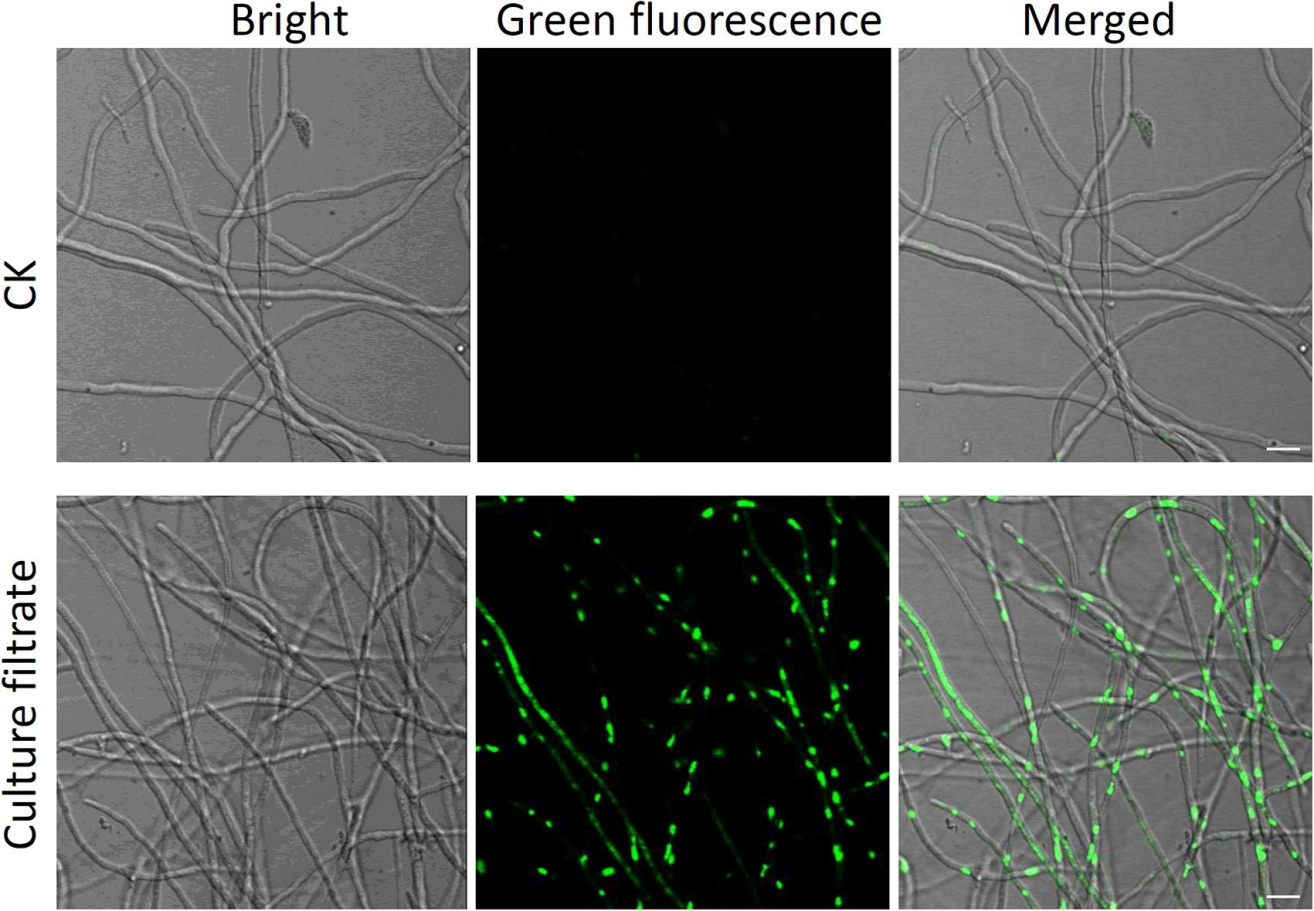
Effect of *B. halotolerans* strain Pl7 culture filtrate against the membrane permeability of *B. dothidea* hyphae, as demonstrated by SYTOX green staining. Bar = 10 μm.

SYTOX green staining was applied to determine whether *B. halotolerans* strain Pl7 affected the permeabilization of *B. dothidea* cell plasma membrane. The result of GFP fluorescence observation showed that no obvious GFP fluorescent signal was observed in CK-treated *B. dothidea* hyphae (Figure 6). In contrast, a bright GFP fluorescent signal was detected in hyphae after *B. halotolerans* strain Pl7 culture filtrate treatment (Figure 6). These results indicated that culture filtrate of *B. halotolerans* strain Pl7 could cause cell membrane permeabilization of *B. dothidea*.

### Antifungal activity of *Bacillus halotolerans* strain Pl7 against apple ring rot

**Figure 7:**
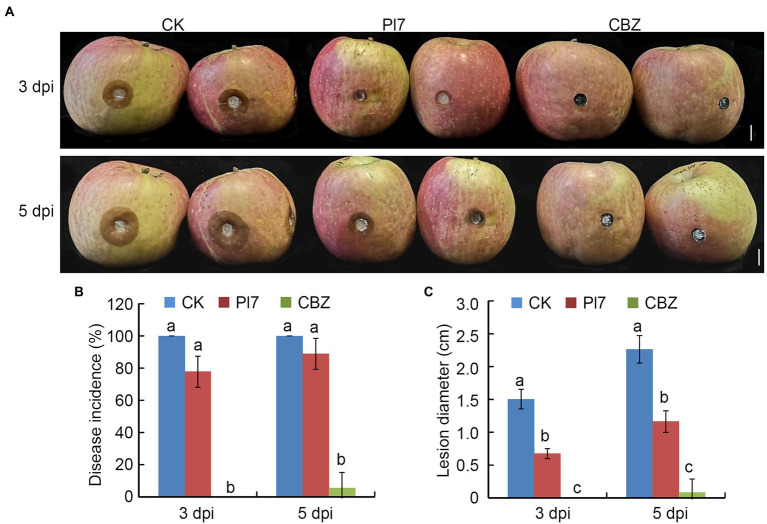
Antifungal activity of *B. halotolerans* strain Pl7 prevents apple ring rot. **(A)** Controlling apple ring rot by strain Pl7 cell suspension. CBZ treatment was employed as positive control. Bar, 1 cm. **(B)** Disease incidence. Each treatment consisted of five fruits with 10 inoculation sites. At 3 and 5 dpi, the number of diseased sites were recorded. Disease incidence (%) represents the percentage of the number of diseased sites out of total treated sites. Data are presented as the mean ± SD of three independent replicates. **(C)** Disease lesion size. Data are presented as the mean ± SD of three biological replicates. Different lowercase letters represent significantly different means at *p* < 0.05 (Duncan’s multiple test). This experiment was performed independently three times with similar results.

Figure 7A showed that strain Pl7 treatment significantly suppressed the disease development of apple ring rot caused by *B. dothidea*, as compared with the CK treatment. CBZ treatment as a positive control remained disease-free (Figure 7A). Even though there was no discernable difference in disease incidence between the strain and CK treatments (Figure 7B), the average lesion diameter of fruit treated with strain Pl7 was 0.67 cm and 1.16 cm at 3 or 5 dpi, which was much smaller than CK (Figure 7C). Therefore, *B. halotolerans* strain Pl7 treatment effectively inhibited the development of ring rot in the apple fruit.

**Figure 8:**
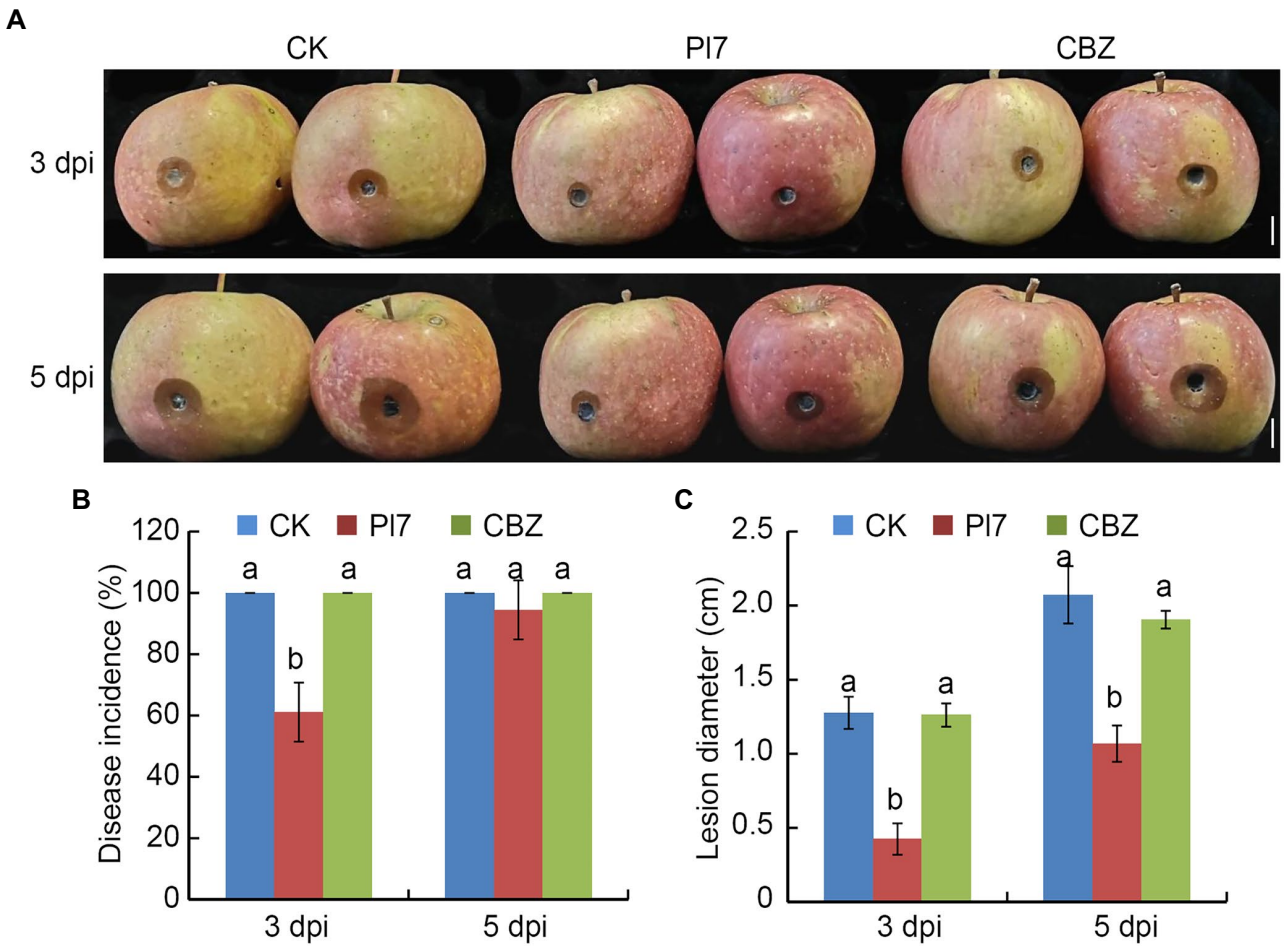
Antifungal activity of *B. halotolerans* strain Pl7 prevents CBZ-resistant isolate-caused apple ring rot. **(A)** Controlling apple ring rot by strain Pl7 cell suspension. Bar, 1 cm. **(B)** Disease incidence. Data are presented as the mean ± SD of three independent replicates. **(C)** Disease lesion size. Data are presented as the mean ± SD of three biological replicates. Different lowercase letters represent significantly different means at *p* < 0.05 (Duncan’s multiple test). This experiment was performed independently three times with similar results.

We also tested the capacity of *B. halotolerans* strain Pl7 to prevent the growth of ring rot in apple fruit caused by a CBZ-resistant isolate. Results indicated that strain Pl7 cell suspension treatment dramatically reduced the average lesion diameter in apple fruit infected with the CBZ-resistant isolate (Figure 8). CBZ treatment did not inhibit CBZ-resistant isolate-caused apple ring rot (Figure 8). At 3 dpi or 5 dpi, the average lesion diameter of CK was 1.28 cm or 2.07 cm, while the strain Pl7-treated fruits was significantly lower (0.42 cm or 1.07 cm; Figure 8C). These data revealed that *B. halotolerans* strain Pl7 was similarly effective in preventing CBZ-resistant isolate-caused apple ring rot.

### Antifungal activity of *Bacillus halotolerans* strain Pl7 against apple postharvest decay

**Figure 9:**
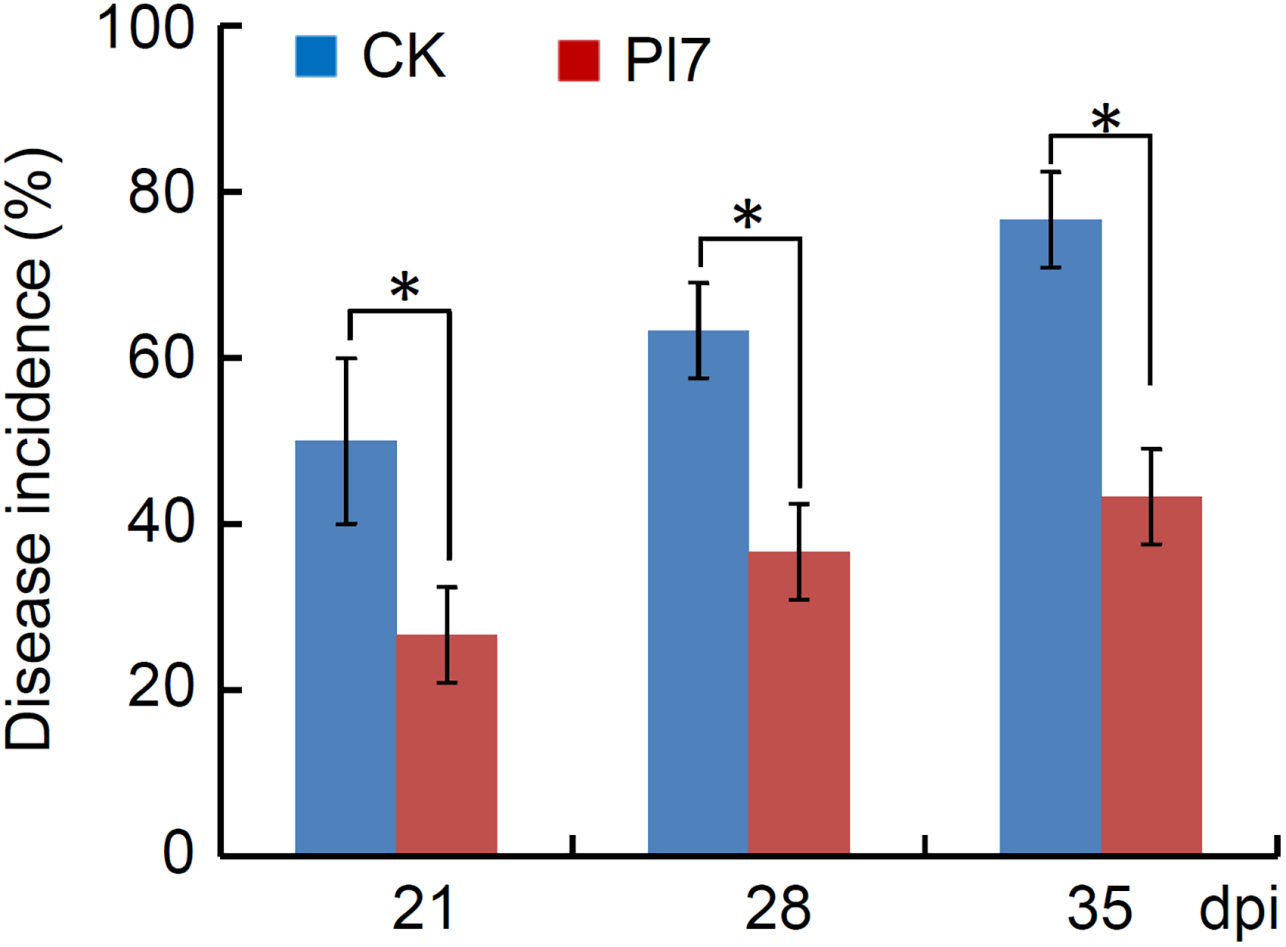
Statistical analysis of disease incidence of *B. halotolerans* strain Pl7-treated apple fruits. After treatment with strain Pl7, the number of rotted fruits was recorded at different storage time points (21, 28, 35 days). Each treatment contained of three replicates and each replicate consisted of 10 fruits. Disease incidence (%) represents the percentage of the number of rotted apple fruits out of total treated apple fruits. Data are presented as the mean of disease incidence ± SD of three biological replicates. Asterisks above the bars represent significantly different (*p* < 0.05; Student’s *t*-test) between CK and treatment. This experiment was performed independently two times with similar results.

During apple storage period, the incidence of apple decay was significantly reduced after strain Pl7 treatment, as compared to CK (Figure 9). At 21 days storage period, the disease incidence of strain Pl7 treated-apple fruits was 27%, which was significantly lower than CK (50%). At 35 days storage period, the disease incidence of CK reached to 77%, while the Pl7 treated-apple fruits was still lower with 43% (Figure 9). These results indicated that strain Pl7 was effectively inhibited apple fruit natural decay.

### Colonization of *Bacillus halotolerans* strain Pl7 in apple fruit wounds

**Figure 10:**
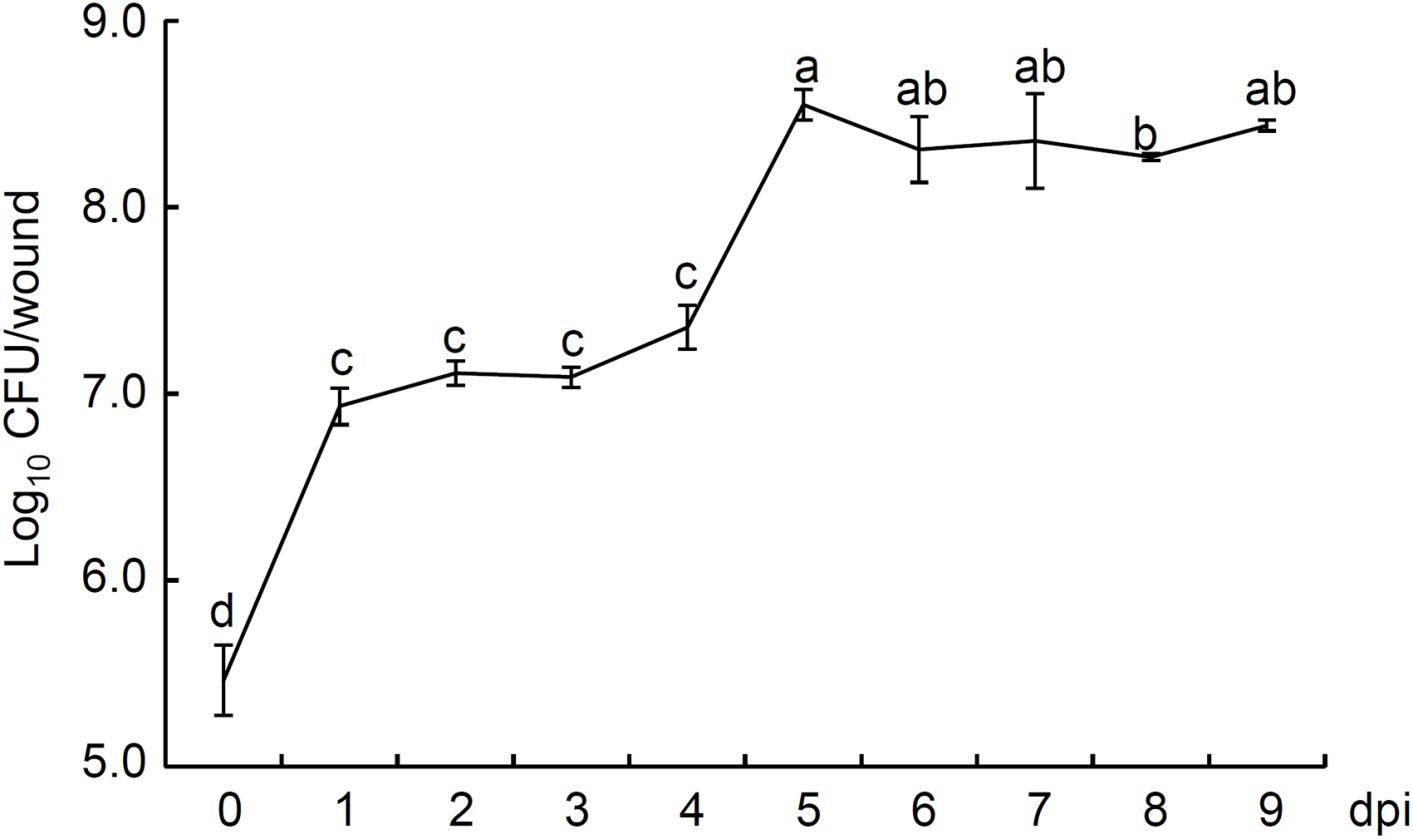
Illustrates the population of *B. halotolerans* strain Pl7 colonies in apple fruit wounds. The colonies were enumerated at the indicated time points, and each time point consisted of three replicates. The population density was converted as Log10 CFU/wound. Data are presented as the mean ± SD of three biological replicates. Different lowercase letters represent significantly different means at *p* < 0.05 (Duncan’s multiple test). The experiment was performed independently two times with similar results.

At 1 dpi, there were 8.57 × 10^6^ CFU of strain Pl7 colonies, which is a 29.54-fold increase from 2.90 × 10^6^ CFU at 0 dpi (Figure 10). At 5 dpi, the number of strain Pl7 colonies reached the peak with the population 3.54 × 10^8^ CFU (Figure 10). After that, the colony number slightly decreased but remained stable (Figure 10). At 9 dpi, strain Pl7 colony number still kept high (2.74 × 10^8^ CFU; Figure 10). These findings suggested that strain Pl7 might successfully colonize apple fruit wounds.

### Transcriptomic analysis of apple fruit

**Figure 11:**
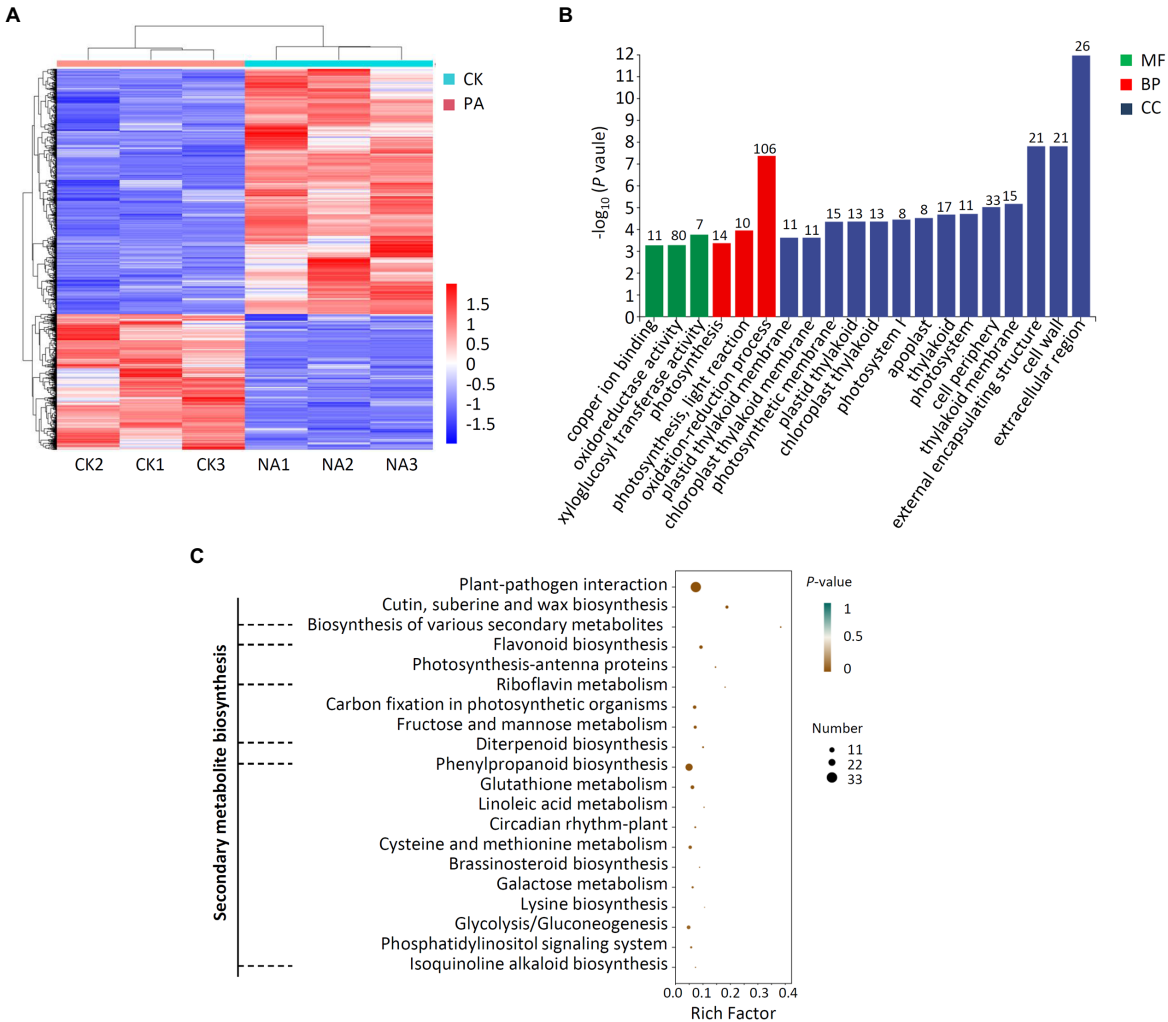
Analysis of DEG transcriptomes in apple fruit treated with *B. halotolerans* strain Pl7. **(A)** Heat map analysis. PA, apple fruit treated with strain Pl7; CK, untreated apple fruit. Gene expression levels are shown in red and blue, respectively. **(B)** DEG annotated GO analysis. The quantity of DEGs was marked above the column. CC, cellular components; BP, biological processes; MP, molecular functions. **(C)** DEG enrichment studies. Rich factor was the DEGs number/total genes found in the transcriptome of a specific activity.

A transcriptome investigation of apple fruit inoculation with *B. halotolerans* strain Pl7 was also carried out to assess overall apple gene expression changes. 489 and 884 DEGs were upregulated and downregulated compared to control samples, respectively (Figure 11A; Supplementary Figure S1). The qRT-PCR result revealed a similar expression pattern of 6 randomly selected genes with RNA-seq (Supplementary Figure S2). This demonstrated the reliability of the transcriptome data used in this study. According to GO analyzes, these DEGs were related to cellular components (CC), especially the extracellular region, cell wall, and cell periphery, biological processes (BP) primarily including oxidation–reduction process, photosynthesis, light reaction, and photosynthesis, molecular functions (MP) primarily including xyloglucosyl transferase activity, oxidoreductase activity and copper ion binding (Figure 11B). Further, KEGG enrichment studies showed that these DEGs were mainly enriched in plant secondary metabolite biosynthesis pathways, such as biosynthesis of various secondary metabolites, flavonoid biosynthesis, riboflavin metabolism, diterpenoid biosynthesis, phenylpropanoid biosynthesis, isoquinoline alkaloid biosynthesis (Figure 11C). In addition, DEGs were also enriched in plant-pathogen interaction (Figure 11C).

Following treatment with strain Pl7, additional analysis showed that 37 DEGs associated with plant secondary metabolism were elevated to varying degrees in apple fruit (Supplementary Table S3). Several potential defense-related genes, such as pathogen-related genes and receptor protein genes, were also greatly induced in apple fruit treated with strain Pl7 (Supplementary Table S4). Taken together, *B. halotolerans* strain Pl7 enhanced apple resistance by inducing genes involved in plant secondary metabolite biosynthesis and plant-pathogen interaction.

### Effect of *Bacillus halotolerans* strain Pl7 on apple postharvest quality

Assays for fruit firmness, ascorbic acid, soluble sugar, and titratable acid revealed no significant differences between apple fruit treated with strain Pl7 and those from the CK group (Table 1). These findings suggested that *B. halotolerans* strain Pl7 had no discernible impact on the apple postharvest quality.

**Table 1:** Effect of *B. halotolerans* strain Pl7 on apple postharvest quality.

Treatment	Firmness (*N*)	Ascorbic acid (mg/100 *g*)	Soluble sugar (%)	Titratable acidity (%)
CK	53.08 ± 3.93	2.27 ± 0.04	8.91 ± 0.30	0.18 ± 0.01
Pl7	50.48 ± 5.14 ns	2.48 ± 0.05 ns	9.63 ± 0.40 ns	0.20 ± 0.01 ns

LB-treated apples were used as CK. Each treatment contained of three replicates and each replicate consisted of three fruits. Data represents the mean ± SD of three biological replicates. ns means no significant difference (*p* < 0.05; Student’s *t*-test) between CK and treatment. This experiment was performed independently two times with similar results.

## Discussion

In this study, *B. halotolerans* strain Pl7 showed strong antagonistic action toward *B. dothidea*. Additionally, *B. dothidea*-induced apple ring rot was considerably reduced by strain Pl7. Interestingly, this strain could similarly reduce the capacity of the CBZ-resistant isolate to produce apple ring rot. This provides us with an alternative method to control the emergence of fungicide-resistant isolates in the future. Taken together, *B. halotolerans* strain Pl7 is a promising biocontrol agent of *B. dothidea*-induced postharvest decay of apples. Previous studies reported the similar results of *B. halotolerans* strains in preventing other fruit postharvest diseases (Slama et al., 2019; Wang et al., 2021). Therefore, *B. halotolerans* strain has the potential to be as commercial biocontrol agent in managing fruit postharvest diseases.

Competition for nutrients and available space with host pathogens is one of the key antagonistic mechanisms for biocontrol agents (Eljounaidi et al., 2016; De Silva et al., 2019). According to this investigation, *B. halotolerans* strain Pl7 was able to quickly colonize and persist in lesions of apple fruit. The population of strain Pl7 colonies significantly expanded, peaked at 5 dpi, and kept high during the following storage period, indicating the quick adaptation of strain Pl7 in the wounds of apple fruit. Other studies have revealed that the capacity of biocontrol agents to colonize and persist in host tissue was essential for their control efficacy (Mahunu et al., 2016; He et al., 2020).

One of the antifungal strategies used by the *Bacillus* species is to inhibit the growth of the pathogen. About 69% of the mycelial growth was considerably inhibited by strain Pl7 *in vitro*. Additional findings indicated that the strain Pl7 culture filtrate mediated antifungal activity against *B. dothidea,* indicating the presence of antimicrobial compounds in the culture filtrate. PCR amplification showed that strain Pl7 harbored gene clusters involved in the synthesis of antimicrobial lipopeptides. Strong hydrolytic enzyme activity including protease, cellulase, and β-1, 3-glucanase was detected in strain Pl7 secretions. These enzymes are cell wall degrading enzymes (CWDEs) that can hydrolyze cell walls of fungi pathogens (Park et al., 2012; Jadhav et al., 2017). Many studies have proved that CWDEs produced by biocontrol agents play an important role in inhibiting the growth of fungi pathogens (Spadaro et al., 2011; Saravanakumar et al., 2016). For example, Zhang et al. (2019a) showed that overexpression of β-glucanase gene *Bgy6* could enchance the control efficacy of endophytic bacteria *B. halotolerans* strain Y6 against *Verticillium dahliae.* In addition, the culture filtrate of strain Pl7 could damage the cell membranes of *B. dothidea*. Cell walls and membranes commonly serve as targets for antifungal treatment because of their important role in fungal growth and response to different environmental stress (Bowman and Free, 2006; Jordá and Puig, 2020). These findings imply that the antifungal action of *B. halotolerans* strain Pl7 against *B. dothidea* growth is relevant to its ability to influence cell wall and cell membrane integrity. A similar antagonistic mechanism was obtained from antifungal peptide of biocontrol agents (Han et al., 2019; Wang et al., 2021).

A series of secondary metabolites enhance the resistance of plants to diseases (Piasecka et al., 2015; Khare et al., 2020). Notably, antagonistic agent treatment could activate the pathway of secondary metabolites biosynthesis, such as phenylpropanoid biosynthesis, in the host plant (Chen et al., 2021; Yuan et al., 2022b). In the present study, apple fruit treated with *B. halotolerans* strain Pl7 produced comparable results. A total of 37 upregulated DEGs in apple fruit were enriched in secondary metabolite biosynthesis pathways. Among them, genes encoding phenylalanine ammonia-lyase, β-glucosidase, 4-coumaroyl-CoA ligase, cinnamoyl-CoA reductase, trans-cinnamate 4-monooxygenase, and peroxidase involved in phenylpropanoid biosynthesis were significantly upregulated by varying degrees. Flavonoid biosynthesis, as a branch of the phenylpropanoid biosynthesis (Khalid et al., 2019), was also enriched in apple fruit treated with strain Pl7, with a significant increase in gene expression of flavanone 3-hydroxylase-like. Lignin is a phenylpropane derivative that strengthens plant cell walls to act as an inducible physical barrier that prevents pathogen penetration (Barros et al., 2015). Caffeoyl shikimate esterase (CSE) is an important enzyme in lignin biosynthesis pathway (Vanholme et al., 2013). We found that homologs of *CSE* were upregulated by 1.34- and 1.23-fold time, respectively. In addition, eight genes encoding cytochrome P450 (CYP450) and seven genes encoding UDP-glycosyltransferase (UGT) related to the biosynthesis of triterpene saponins (Cheng et al., 2020), were higher upregulated by strain Pl7 treatment. These results indicated that *B. halotolerans* strain Pl7 can induce apple defenses primarily *via* regulating the secondary metabolite biosynthesis pathways.

The influence of biocontrol agents on fruit quality is a key factor in determining whether they can be used during post-harvest fruit processing (Osman et al., 2011; Sun et al., 2017). According to a prior study, *B. halotolerans* treatment preserved the nutritional value of strawberry (Wang et al., 2021). In the current study, we found that *B. halotolerans* Pl7 treatment had no discernible effects on the apple fruit quality during storage, indicating its potential for use in preventing apple postharvest decay.

## Conclusion

In conclusion, *B. halotolerans* strain Pl7 is identified as a potential biocontrol agent of controlling *B. dothidea*-caused apple postharvest decay during storage. The present study sheds light on the molecular mechanism of *B. halotolerans* strain against *B. dothidea* and provides an alternative and environment-friendly method to chemical fungicides in controlling apple postharvest decay for apple grower. However, further studies need to be investigated before the application of strain Pl7 in field, such as the harmlessness of *B. halotolerans* strain Pl7 to human’s health and the survival rate of strain Pl7 in adverse environmental conditions should be assessed.

## Data availability statement

The data presented in the study are deposited in NCBI, accession number PRJNA911915.

## Author contributions

HY: project administration, supervision, data collection, and writing – review and editing. MY, BS, ZW, and TH: data collection. JZ, HH, and LW: data analysis. HT: supervision and funding acquisition. All authors contributed to the article and approved the submitted version.

## Funding

This work obtained the financial support of the National Key R&D Program of China (No. 2017YFE0135600), Agricultural Science and Technology Innovation Program (CAAS-ASTIP-2016-RIP), and Central Public-Interest Scientific Institution Basal Research Fund (No. ZGS202110).

## Conflict of interest

The authors declare that the research was conducted in the absence of any commercial or financial relationships that could be construed as a potential conflict of interest.

## Publisher’s note

All claims expressed in this article are solely those of the authors and do not necessarily represent those of their affiliated organizations, or those of the publisher, the editors and the reviewers. Any product that may be evaluated in this article, or claim that may be made by its manufacturer, is not guaranteed or endorsed by the publisher.

## Supplementary material

SUPPLEMENTARY FIGURE S1 Transcriptome pattern in apple fruit treated with *B. halotolerans* Pl7. Red dots mean upregulated DEGs, blue dots mean downregulated DEGs, grey dots mean no DEGs.

SUPPLEMENTARY FIGURE S2 qRT-PCR assay. CK, LB broth-treated apple; PA, strain Pl7-treated apple. Data are presented as the mean ± SD of three replicates. This experiment was performed independently two times with similar results.
