# Supplementary figures and images for: Biocontrol activity of Bacillus halotolerans strain Pl7 against Botryosphaeria dothidea causing apple postharvest decay and potential mechanisms
Source: Front Microbiol. 2023 Jan 4;13:1058167. doi: 10.3389/fmicb.2022.1058167 (PMC9846367; doi:10.3389/fmicb.2022.1058167)

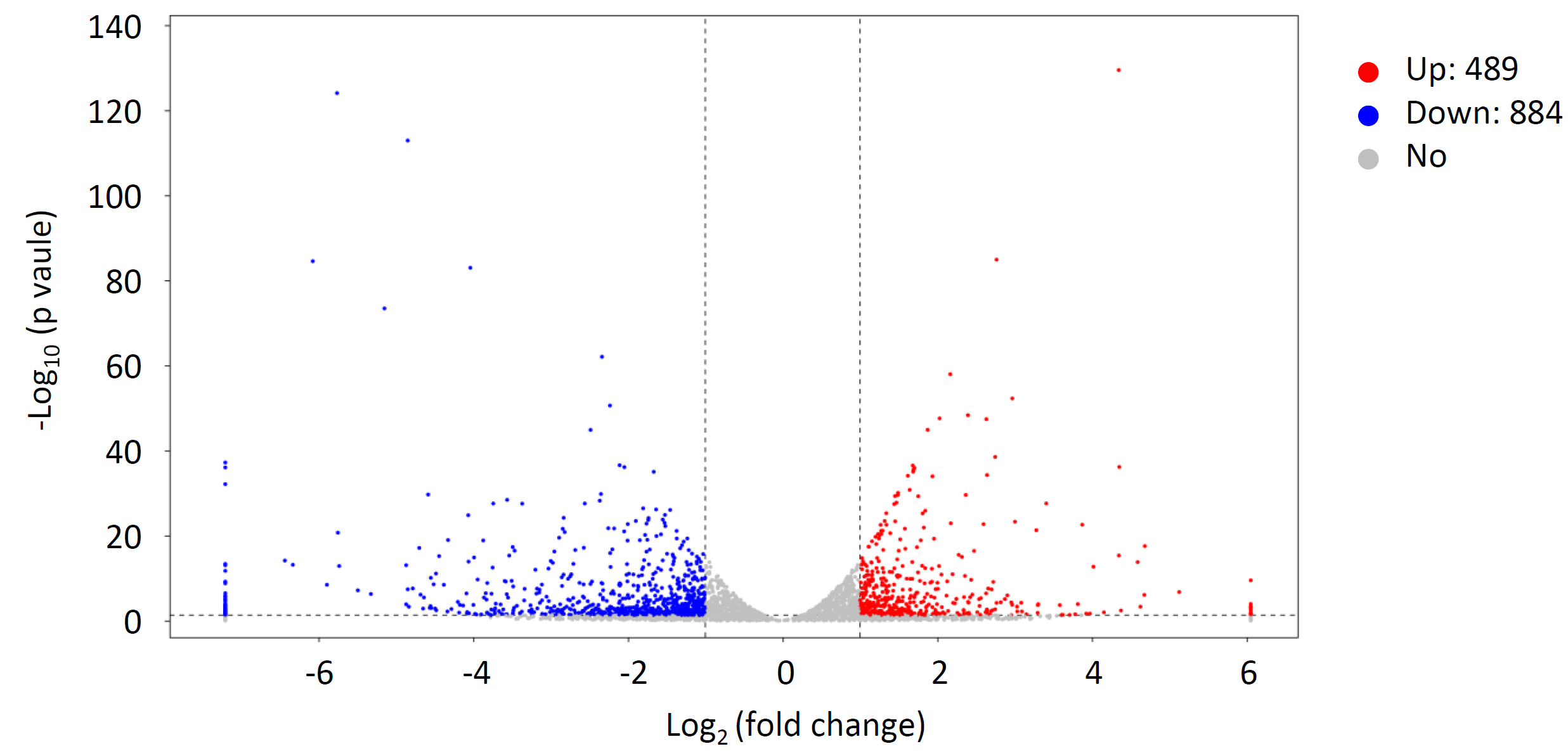

Supplement: SUPPLEMENTARY FIGURE S1 — Transcriptome pattern in apple fruit treated with B. halotolerans Pl7. Red dots mean upregulated DEGs, blue dots mean downregulated DEGs, grey dots mean no DEGs. [file Image_1.TIF]

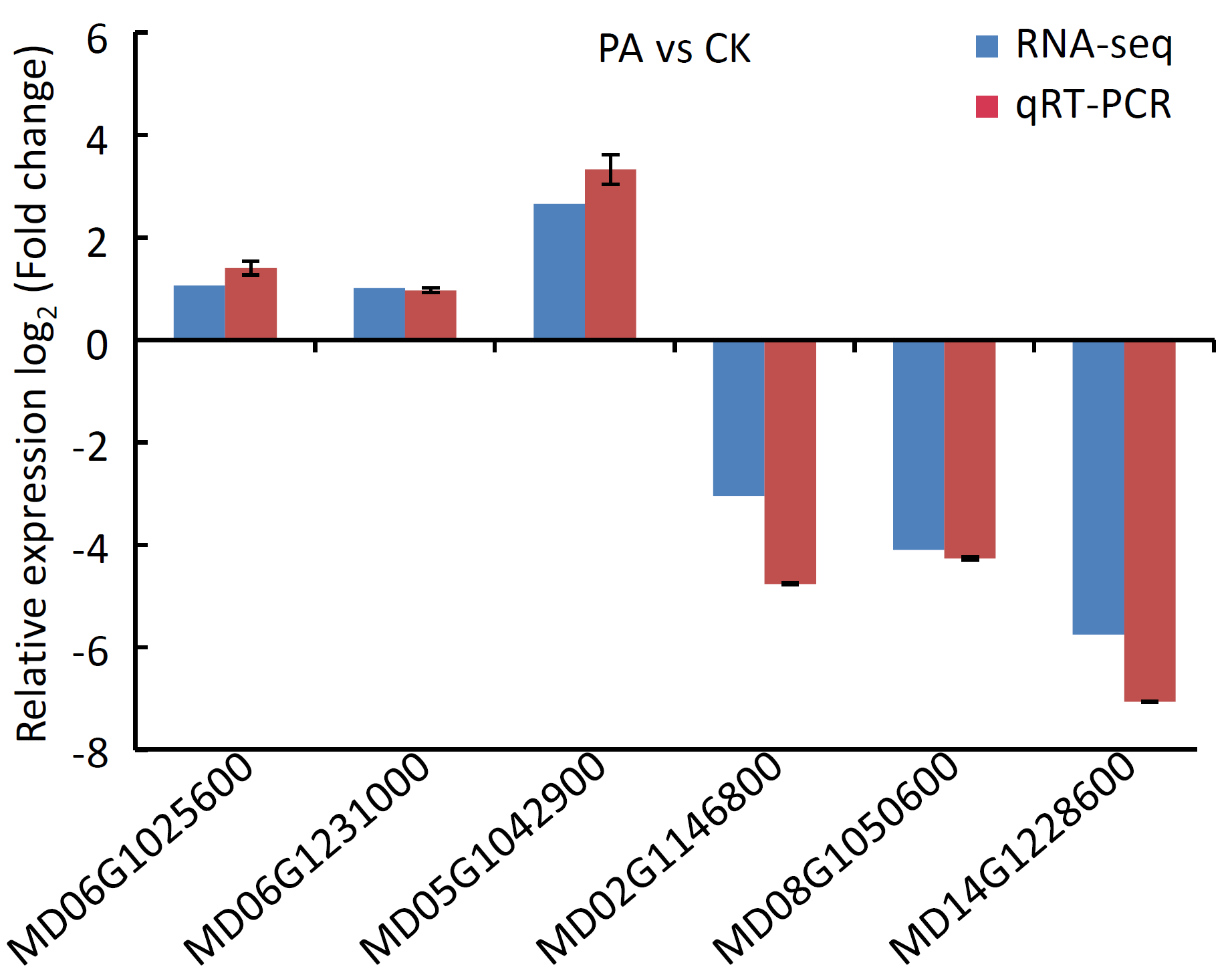

Supplement: SUPPLEMENTARY FIGURE S2 — qRT-PCR assay. CK, LB broth-treated apple; PA, strain Pl7-treated apple. Data are presented as the mean ± SD of three replicates. This experiment was performed independently two times with similar results. [file Image_2.TIF]
